# Comparison of ceftriaxone plus macrolide and ampicillin/sulbactam plus macrolide in treatment for patients with community-acquired pneumonia without risk factors for aspiration: an open-label, quasi-randomized, controlled trial

**DOI:** 10.1186/s12890-020-01198-4

**Published:** 2020-06-05

**Authors:** Nobuyoshi Hamao, Isao Ito, Satoshi Konishi, Naoya Tanabe, Masahiro Shirata, Issei Oi, Mitsuhiro Tsukino, Hisako Matsumoto, Yoshiro Yasutomo, Seizo Kadowaki, Toyohiro Hirai

**Affiliations:** 1 Department of Respiratory Medicine, Graduate School of Medicine, Kyoto University, 54 Shogoin-kawaracho, Sakyo, Kyoto, 606-8507 Japan; 2 Department of Internal Medicine, Ono Municipal Hospital, 323 Naka-cho, Ono, Hyogo 675-1332 Japan; 3 Department of Respiratory Medicine, Hikone Municipal Hospital, 1882 Hassakacho, Hikone, Shiga 522-8539 Japan

**Keywords:** Pneumonia, ABPC/SBT, CTRX

## Abstract

**Background:**

Ceftriaxone (CTRX) and ampicillin/sulbactam (ABPC/SBT) are recommended by various guidelines as the first-line antibiotics for community-acquired pneumonia (CAP). However, which of these antibiotics is more effective for treating non-aspiration CAP remains unclear.

**Methods:**

This study was a prospective, single-center, open-label, quasi-randomized controlled trial. Patients with adult CAP without risk for aspiration were allocated to either a CTRX or ABPC/SBT group based on the date of hospital admission. Macrolide was added to patients in each group. The primary outcome was the clinical response in the validated per-protocol (VPP) population at end of treatment (EOT). The secondary outcomes were clinical response during treatment and at end of study (EOS) in the VPP population, and mortality rate at day 30 in the modified intention-to-treat (MITT) population.

**Results:**

Of 696 screened patients, 433 patients were excluded and 263 patients were allocated to receive either of the treatments. Males comprised 54% of patients and mean age and PSI were 62.1 ± 19.8 years and 69.3 ± 30.0, respectively, with 124 patients allocated to the CTRX group and 138 patients allocated to the ABPC/SBT group. The clinical effectiveness rate for the VPP population at EOT was 90% in the CTRX and 96% in the ABPC/SBT group (*p* = 0.072, 95% confidence interval [CI] of risk difference [RD]: − 12.6–0.8%). No significant difference in effectiveness at day 4 was observed between the CTRX and ABPC/SBT groups (*p* = 0.079, 95%CI of RD: − 12.1–0.4%), but at day 7, ABPC/SBT was significantly more effective than CTRX in the VPP population (*p* = 0.047, 95%CI of RD: − 13.3–-0.4%). No significant difference in late response at EOS was seen between CTRX and ABPC/SBT groups: cure (89 [86%] and 102 [94%]), relapse (5 [5%] and 1 [1%]) and failure (10 [10%] and 5 [5%]; *p* = 0.053). Deaths within 30 days in MITT population was higher in CTRX group (4 [3%]) than in ABPC/SBT group (0 [0%]) (*p* = 0.048, 95%CI of RD: 0.1–6.3%).

**Conclusion:**

No significant difference in effectiveness was found between ABPC/SBT and CTRX at EOT. However, ABPC/SBT might be more effective in the early phase of treatment.

**Trial registration:**

UMIN-CTR, UMIN000037464. Registered 25 July 2019 – Retrospectively registered, https://upload.umin.ac.jp/cgi-open-bin/ctr_e/ctr_view.cgi?recptno=R000042262

## Background

Pneumonia is caused by a wide variety of pathogens, and is one of the most common infections around the world. Community-acquired pneumonia (CAP) is caused by bacteria such as *Streptococcus pneumoniae*, *Haemophilus influenzae*, and *Moraxella catarrhalis* or viruses such as influenza virus [1, 2]. Susceptibility to antibiotics varies depending on the pathogen. For example, the susceptibilities of *Streptococcus pneumoniae*, *Haemophilus influenzae* and methicillin-resistant *Staphylococcus aureus* to β-lactam/β-lactamase inhibitors were reported as 99.5%, 59.3–78.0% and 7.7–20.2%, and the susceptibilities of these species to third-generation cephalosporins were reported as 96.8, 100 and 1.0% [1]. Antibiotics were therefore selected on the basis of presumptive bacteria in consideration of the patient’s age, comorbidities, symptoms, laboratory findings, severity, and so on.

Ceftriaxone (CTRX) and ampicillin/sulbactam (ABPC/SBT) are recommended by various guidelines for pneumonia in a number of countries as the first-line antibiotics for CAP [3–7]. Both of these antibiotics are active against a similar range of microorganisms, except for anaerobic bacteria, which are the predominant pathogens in aspiration pneumonia [8–10]. Because the susceptibility of anaerobic bacteria to CTRX is relatively low [11, 12], some guidelines recommend ABPC/SBT for the treatment of aspiration pneumonia [3, 4]. In previous reports that did not consider aspiration risks, no significant differences were found between CTRX and β-lactam/β-lactamase inhibitor combinations such as ABPC/SBT for the treatment of pneumonia [13–16]. Only one paper has compared ABPC/SBT with CTRX in the treatment of aspiration pneumonia patients [17]. However, that study was a retrospective propensity score-matching analysis and whether this result is applicable to aspiration pneumonia in clinical practice remains unclear.

Some reports have described CAP excluding aspiration pneumonia [18], but most previous studies of antibiotic treatments for CAP have included patients with aspiration pneumonia. To our knowledge, no reports have compared CTRX and ABPC/SBT for the treatment of CAP in patients without risk factors for aspiration. We therefore carried out the present study with the aim of investigating whether CTRX might be more effective than ABPC/SBT for the treatment of CAP, after excluding cases of aspiration pneumonia.

## Methods

### Patients

We enrolled patients aged ≥15 years who had been hospitalized with CAP without risk factors for aspiration, as described in our previous study [19]. The diagnostic criteria for CAP are defined as radiological findings of a new and/or progressive infiltrate(s) and two or more of the following symptoms: cough, sputum or change of sputum character (increased volume and/or purulence), dyspnea, pleuritic chest pain, tachycardia, documented axillary body temperature ≥ 37.5 °C within the past 24 h, rigors and/or chills, general malaise, abnormal breathing sounds, auscultatory findings consistent with the lung infiltrate on chest examination, and white blood cell (WBC) count < 3000/mm^3^ or ≥ 10,000/mm^3^. Severity of pneumonia was determined according to the pneumonia severity index (PSI) [20].

Cases meeting any of the following criteria were excluded: suspected aspiration pneumonia or hospital-acquired pneumonia; hospitalization within 60 days of symptom onset; active lung cancer (cases other than completely resected ones); terminal illness; immunocompromising disease (human immunodeficiency virus infection, active hematologic malignancies, neutropenia and congenital immunodeficiency) or receipt of immunosuppressive therapy (use of ≥10 mg of prednisolone-equivalents, and/or immunosuppressants); pregnant or breastfeeding; known allergy to the indicated antibiotics; or presence of other infiltrative diseases such as organizing pneumonia, radiation pneumonitis, drug-induced pneumonia, obstructive pneumonia, tuberculosis or fungal infection, and empyema.

To judge whether a case represented suspected aspiration pneumonia, patients were evaluated for various aspiration risk factors [21–27], including the following: neurological disorders such as cerebrovascular disease, neuromuscular disease, and dementia; oral/pharyngeal/throat disorders; bedridden state; gastroesophageal disorders such as gastroesophageal reflux disease, esophageal diverticulum, esophageal cancer, achalasia, systemic sclerosis, post-gastrectomy (total or partial), and hiatal hernia; insertion of a nasogastric tube; currently taking sedatives or hypnotics; subjective or observed aspiration/choking/dysphagia; or episodes of vomiting. Patients having one or more of these risk factors were excluded.

### Setting and design

This prospective, single-center, open-label, quasi-randomized study was conducted from June 3, 2002 to June 30, 2008 at Ono Municipal Hospital (Ono, Hyogo, Japan). The institutional review board of Ono Municipal Hospital approved the study protocol, and written, informed consent was obtained from all patients. If patient was under 20 years, the consent of his/her parent was also obtained. Patients were enrolled and allocated by attending doctor. Treatment allocation was performed based on the day of month on which the patient was admitted to hospital. If the day of month of admission was an odd number, patients were treated using intravenous CTRX at 2 g every 24 h. If the day of month of admission was an even number, patients were treated using intravenous ABPC/SBT at 3 g every 12 h. These dosages were determined based on drug information available in Japan at the launch of the study. The dose of CTRX or ABPC/SBT was adjusted as follows based on the creatinine clearance rate (CCr): CCr 10–50 mL/min, CTRX 1 g every 24 h or ABPC/SBT 3 g every 12 h; and CCr < 10 mL/min, CTRX 1 g every 24 h or ABPC/SBT 1.5 g every 24 h. In patients with mild to moderate pneumonia (PSI I–IV), clarithromycin 200 mg was given orally every 12 h in addition to CTRX or ABPC/SBT. This dosage was determined because only clarithromycin 200 mg tablets could be used in Japan and this dosage was approved dose at the time of study. In patients with severe pneumonia (PSI V), erythromycin 500 mg was given intravenously every 6 h in addition to CTRX or ABPC/SBT. No other antibiotics were allowed. The patients received antibiotic treatment for 7–14 days, until their body temperature was < 37 °C for 48 h with clinical stability, and improvements were seen in terms of dyspnea, sputum, or C-reactive protein (CRP) levels. When a patient showed a recurrence of fever > 37.5 °C after initial improvement of fever, the same antibiotic therapy was continued for 4 days from the first day of recurrence.

### Clinical and bacteriological evaluations

The following clinical data were evaluated on admission: vital signs, comorbidities, physical examination findings, prior antibiotic treatment, and allergy to antibiotics. To evaluate the effects of treatment, clinical findings, chest radiography findings, and laboratory test results were collected before, during, and at end of treatment (EOT; days 7–14). The late response to treatment was evaluated at end of study (EOS; days 28–35).

All patients underwent a microbiological evaluation before starting treatment with antibiotics. Sputum specimens were obtained for Gram staining and cultures if possible. Blood specimens were obtained for cultures. Urine specimens were tested for urinary antigens of *S. pneumoniae* (BinaxNOW® *S. pneumoniae* urinary antigen test; Inverness Medical Innovations, Waltham, MA, USA) and *Legionella pneumophilia* serogroup 1 (BinaxNOW® *Legionella* urinary antigen test). *Mycoplasma pneumoniae* antibody was tested using a paired particle hemagglutinin test, and *Chlamydophila pneumoniae* antibody using a paired enzyme-linked immunosorbent assay (Hitazyme; Hitachi Chemical, Tokyo, Japan). Influenza virus antigen was tested using throat or nasal swab specimens between November and March.

### Criteria for evaluation

The primary outcome was the clinical response at EOT in the validated per-protocol (VPP) population. The secondary outcomes were clinical response during treatment (at days 4 and 7) and at EOS in the VPP population, and the mortality rate at 30 days of admission in the modified intention-to-treat (MITT) population. The VPP population was defined as patients who received treatment with the study drug for ≥4 days in cases of clinical cure, or ≥ 3 days in cases of clinical failure without a protocol violation or missing data. The following patients were excluded: those lacking information or clinical data; those treated with other antibiotics in addition to the study drug; and those treated with systemic corticosteroids affecting the judgment of the effectiveness of treatment in the VPP population. All patients treated with one or more doses of the study drug were included in the MITT population.

The effectiveness of treatment was assessed by blinded investigators. The assessment was based on clinical signs and symptoms, WBC count, serum CRP, and chest radiography. Serum CRP and chest radiography were not used for the assessment on day 4, because the findings might appear to be worse than those on day 1 despite an improvement in patient condition. The clinical response was classified into three categories: improving, indeterminate, or worsening. The test drugs were discontinued and changed to other antibiotics when the clinical response was judged to fall in the latter two categories. “Effective” on days 4, 7 and EOT was defined as a case in which the clinical response was judged as improving at the respective time point. Late response at EOS was classified into three categories: cure, relapse, and failure. Patients with resolution of signs and symptoms of pneumonia were classified as “cure,” whereas those with recurrent fever related to pneumonia after day 7, after being primarily judged as improving by EOT, were classified as “relapse,” and those without improvement of signs and symptoms of pneumonia or with need for alteration of antibiotics were classified as “failure.”

### Statistical analysis

The normally distributed data were evaluated using Student’s *t*-test, and are reported as mean ± standard deviation (SD). Non-normally distributed data were evaluated using the Mann-Whitney *U* test, and are reported as median (interquartile range). Differences in population rates between the two or more groups were evaluated using the chi-squared test. The two-tailed significance level was set at < 0.05. Sample size was calculated as follows. If the average clinical effectiveness rate at EOT was estimated to be ≥90%, and the difference in clinical effectiveness rate to be ≥12% based on previous reports [13, 28], the sample size required to detect a difference with a β value < 0.20 was 192 cases. If the rate of excluded cases was assumed to be < 30%, 274 CAP cases would need to be screened. All statistical tests were performed using JMP 14.0.0 (SAS Institute Inc., Cary, NC).

## Results

### Patients

**Fig. 1:**
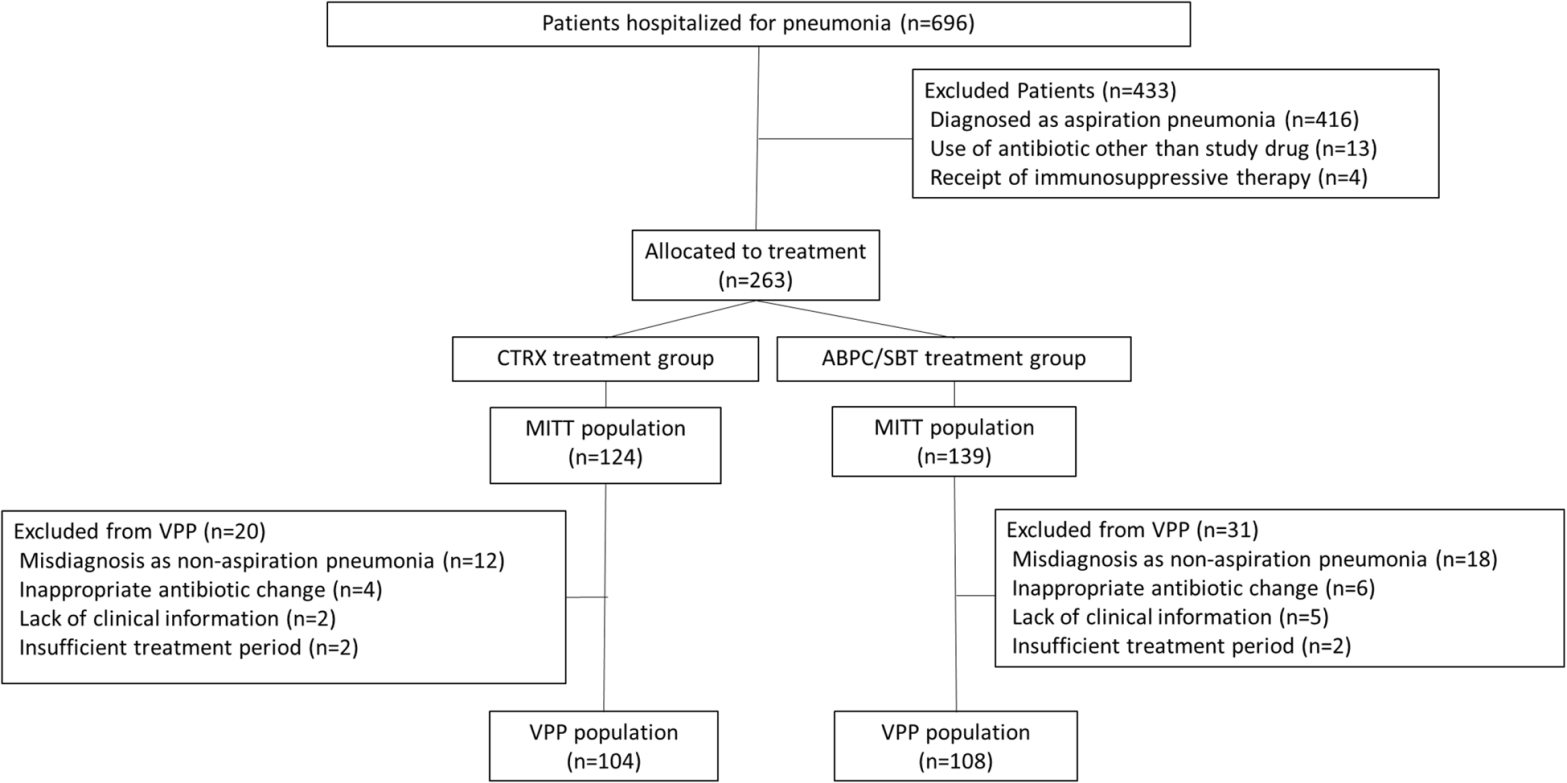
Flowchart of study enrollment. Four hundred thirty-three patients were excluded from analysis because of aspiration risks (*n* = 416), a protocol violation by a physician (*n* = 13), and the use of immunosuppressive drugs (*n* = 4). CTRX = ceftriaxone; ABPC/SBT = ampicillin/sulbactam; MITT = modified intention-to-treatment; VPP = validated per protocol

Patient recruitment ended June 30, 2008 as planned. The last patient was followed up until July 8, 2008. A total of 696 patients were hospitalized for pneumonia during the study period. Among these, 433 patients were excluded: 416 because of aspiration risks, 13 because of a protocol violation by a physician, and four because of receipt of immunosuppressive therapy. Therefore, 263 patients were finally included in this study (Fig. 1), among whom, 124 were allocated to the CTRX group, and 139 to the ABPC/SBT group. In two cases from the CTRX group and three cases from the ABPC/SBT group, no macrolides were administered. Fifty-one patients were excluded from the VPP population: 30 because of misdiagnosis with non-aspiration pneumonia, 10 because of inappropriate antibiotic change, seven because of a lack of information, and four because of insufficient treatment period. No significant differences in the numbers of cases excluded from the VPP were observed between the two groups. Baseline clinical characteristics of the MITT and VPP populations are summarized in Tables 1 and 2; these characteristics were similar between the two treatment groups except for CRP, which was higher in the ABPC/SBT group than in the CTRX group in the MITT population, but did not differ significantly between the two groups in the VPP population. This study included patients with low PSI, due to the universal health-care coverage in Japan. Patients with low PSI are thus more likely to be hospitalized in Japan than in other countries.

**Table 1:** Baseline characteristics of patients in the modified intention-to-treat (MITT) population

	CTRX (*n* = 124)	ABPC/SBT (*n* = 139)	*p*-value
Male/female, n (% male)	63/61 (51)	80/59 (58)	0.32*
Age, y	60.6 ± 21.3	63.4 ± 18.4	0.25
Comorbidities			
Cancer	10 (8)	14 (10)	0.67*
Hematological malignancy	2 (2)	0 (0)	0.22^†^
Cardiovascular disease	8 (6)	14 (10)	0.37*
Chronic lung disease	23 (19)	24 (17)	0.87*
Bronchial asthma	11 (9)	13 (9)	1.00*
COPD	7 (6)	8 (6)	1.00*
Chronic liver disease	2 (2)	1 (1)	0.60^†^
Chronic kidney disease	3 (2)	2 (1)	0.67^†^
Pneumonia severity index	67.8 ± 32.6	70.7 ± 27.5	0.45
Class I, n (%)	29 (23)	24 (17)	0.71^‡^
Class II, n (%)	40 (32)	47 (34)	
Class III, n (%)	25 (20)	44 (32)	
Class IV, n (%)	24 (19)	20 (14)	
Class V, n (%)	6 (5)	4 (3)	
Performance status, n (%)			
0	108 (87)	122 (88)	0.87^‡^
1	11 (9)	12 (9)	
2	4 (3)	2 (1)	
3	0 (0)	2 (2)	
4	1 (1)	1 (1)	
Maximum body temperature, °C			
Before treatment	38.1 ± 0.8	38.1 ± 1.0	0.89
Day of visit	38.0 ± 0.8	38.0 ± 1.0	0.79
Systolic blood pressure, mmHg	127.8 ± 21.5	131.4 ± 24.8	0.22
Diastolic blood pressure, mmHg	73.0 ± 15.9	73.1 ± 13.1	0.97
Pulse rate, beats/min	90.3 ± 16.7	89.0 ± 14.9	0.49
Respiration rate, breaths/min (n)	21.2 ± 4.6 (83 cases)	22.2 ± 5.5 (99 cases)	0.18
CRP, mg/dL	10.7 ± 7.2	12.9 ± 7.7	0.02
WBC, ×10^3^/μL	11.6 ± 5.2	11.3 ± 5.6	0.60
Albumin, g/dL (n)	3.8 ± 0.5 (123 cases)	3.7 ± 0.4 (137 cases)	0.42

*CTRX* Ceftriaxone, *ABPC/SBT* Ampicillin/sulbactam, *CRP* C-reactive protein, *WBC* White blood cell. Data are presented as mean ± standard deviation (SD) for continuous variables and number (%) for categorical variables. Comparisons were conducted using Student’s *t*-test unless otherwise indicated. *Chi-squared test. ^†^Fisher’s exact test. ^‡^Mann-Whitney *U* test

**Table 2:** Baseline characteristics of patients in the validated per protocol (VPP) population

	CTRX (*n* = 104)	ABPC/SBT (*n* = 108)	*p*-value
Male/female, n (% male)	55/49 (53)	60/48 (56)	0.78*
Age, y	59.0 ± 21.6	63.2 ± 18.9	0.13
Comorbidities*			
Cancer	8 (8)	12 (11)	0.48*
Hematological malignancy	2 (2)	0 (0)	0.24^†^
Cardiovascular disease	7 (7)	12 (11)	0.34*
Chronic lung disease	21 (20)	19 (18)	0.73*
Bronchial asthma	11 (11)	10 (9)	0.82*
COPD	6 (6)	6 (6)	1.00*
Chronic liver disease	1 (1)	0 (0)	0.49^†^
Chronic kidney disease	2 (2)	2 (2)	1.00^†^
Pneumonia severity index	66.3 ± 32.5	68.5 ± 26.7	0.60
Class I, n (%)	26 (25)	20 (19)	0.63^‡^
Class II, n (%)	35 (34)	36 (33)	
Class III, n (%)	20 (19)	38 (35)	
Class IV, n (%)	19 (18)	11 (10)	
Class V, n (%)	4 (4)	3 (3)	
Performance status, n (%)			
0	93 (89)	98 (91)	0.76^‡^
1	9 (9)	8 (7)	
2	2 (2)	1 (1)	
3	0 (0)	1 (1)	
4	0 (0)	0 (0)	
Maximum body temperature, °C			
Before treatment	38.1 ± 0.9	38.1 ± 1.0	0.77
Day of visit	38.0 ± 0.9	38.1 ± 1.0	0.77
Systolic blood pressure, mmHg	127.8 ± 20.9	132.0 ± 24.4	0.18
Diastolic blood pressure, mmHg	73.0 ± 16.2	73.3 ± 13.4	0.90
Pulse rate, beats/min	91.2 ± 17.2	89.2 ± 15.8	0.36
Respiration rate, breaths/min (n)	21.1 ± 4.9 (68)	22.0 ± 5.3 (77)	0.31
CRP, mg/dL	11.0 ± 7.1	12.5 ± 7.5	0.12
WBC, ×10^3^/μL	11.7 ± 4.9	11.0 ± 5.3	0.37
Albumin, g/dL (n)	3.8 ± 0.5 (103)	3.8 ± 0.4 (106)	0.97

*CTRX* Ceftriaxone, *ABPC/SBT* Ampicillin/sulbactam, *CRP* C-reactive protein, *WBC* White blood cell. Data are presented as mean ± standard deviation (SD) for continuous variables and number (%) for categorical variables. Comparisons were conducted using Student’s *t*-test unless otherwise indicated. *Chi-squared test. ^†^Fisher exact test. ^‡^Mann–Whitney *U* test

### Clinical outcomes

**Fig. 2:**
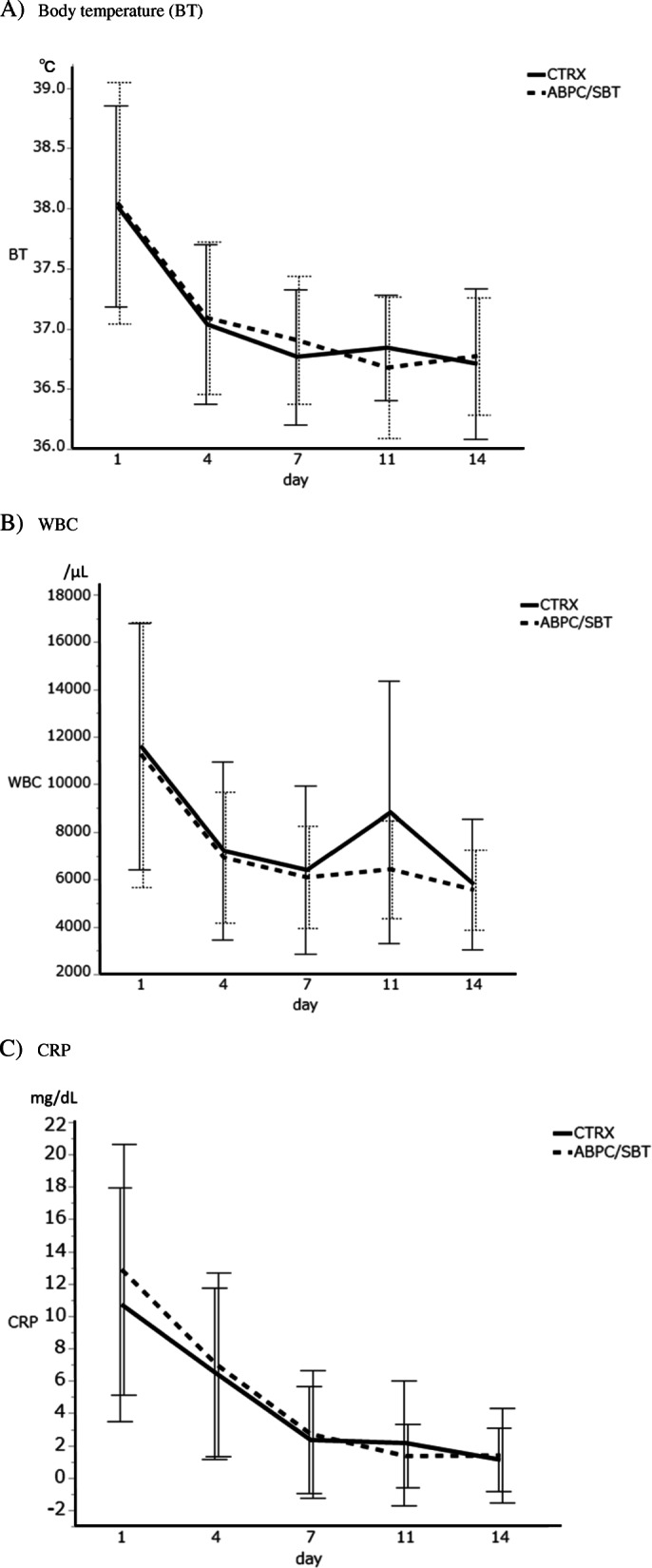
Time-course analyses of body temperature (**a**), white blood cell (WBC) count (**b**), and C-reactive protein (CRP) levels (**c**) in the modified intention-to-treatment (MITT) population. Data are expressed as mean ± standard deviation (SD). The solid line shows ceftriaxone (CTRX), and the broken line shows ampicillin/sulbactam (ABPC/SBT). No significant difference was seen between the two groups in BT, WBC count or CRP levels by paired *t*-test

The primary and secondary outcomes are shown in Table 3. The clinical effectiveness rate for the VPP population at EOT was 90% in the CTRX group and 96% in the ABPC/SBT group, indicating a marginal difference between the two groups (*p* = 0.072). In addition, in the VPP population, the effectiveness rate at day 7 was significantly higher in the ABPC/SBT than in the CTRX group (*p* = 0.047). In the MITT population, effectiveness rates on days 4 and 7 were significantly higher in the ABPC/SBT group than in the CTRX group (*p* = 0.018, *p* = 0.007). Time-course analyses of axillary temperature, WBC count, and CRP level in the MITT population showed no significant differences between the two groups (Fig. 2).

**Table 3:** Clinical outcomes in the modified intention-to-treatment (MITT) and validated per protocol (VPP) populations

	CTRX	ABPC/SBT	Risk Difference % (95% CI)	*p*-value
MITT population, n	124	139		
Treatment period, days±SD	7.8 ± 3.2	8.2 ± 3.7		0.245*
Day 4, effective, n (%)	106 (88) (121 cases)	130 (96) (135 cases)	−8.7 (−15.4–-2.0)	0.011
Day 7, effective, n (%)	105 (88) (120 cases)	126 (97) (130 cases)	−9.4 (−16.0–-2.8)	0.007
EOT, effective, n (%)	108 (87)	130 (94)	−7.1 (−14.2–0.0)	0.055
EOS, cure, n (%)	102 (83)	126 (91)		0.187
EOS, relapse, n (%)	5 (4.0)	3 (2)		
EOS, failure, n (%)	17 (14)	10 (7)		
Death within 30 days, n (%)	4 (3)	0 (0)	3.2 (0.1–6.3)	0.048^†^
VPP population, n	104	108		
Treatment period, days±SD	7.9 ± 2.9	8.3 ± 3.3		0.387*
Day 4, effective, n (%)	95 (91)	105 (97)	−5.9 (−12.1–0.4)	0.079^†^
Day 7, effective, n (%)	94 (90)	105 (97)	−6.8 (−13.3–-0.4)	0.047^†^
EOT, effective, n (%)	94 (90)	104 (96)	−5.9 (−12.6–0.8)	0.072^†^
EOS, cure, n (%)	89 (86)	102 (94)		0.053
EOS, relapse, n (%)	5 (5)	1 (1)		
EOS, failure, n (%)	10 (10)	5 (5)		
Death within 30 days, n (%)	1 (1)	0 (0)	1.0 (−0.9–2.8)	0.491^†^

*CTRX* Ceftriaxone, *ABPC/SBT* Ampicillin/sulbactam, *CI* Confidence interval, *EOT* End of treatment, *EOS* End of study. Relapse was defined as cases with recurrent fever after day 7 after primarily being judged as improving by EOT. Comparisons were conducted using the chi-squared test unless otherwise indicated. *Student’s *t*-test. ^†^Fisher’s exact test

### Bacteriologic analysis

The presumptive pathogen was identified in 136 (52%) of the 263 patients (Table 4). The most common pathogen was *S. pneumoniae* (35 patients [28%] in the CTRX group and 26 [19%] in the ABPC/SBT group). Of these, eight patients in the CTRX group and one in the ABPC/SBT group appeared to have mixed infections with other pathogens. In addition, 38 and 32 atypical pneumonia cases were seen in the CTRX and ABPC/SBT groups, respectively. Viral pneumonia was seen in two cases in the CTRX and one case in the ABPC/SBT group.

**Table 4:** Presumptive causative pathogens in the two treatment groups

	CTRX *n* = 124	ABPC/SBT *n* = 139
*Streptococcus pneumoniae*	35 (10)	26 (1)
*MSSA*	0	2
*Haemophilus influenzae*	3 (1)	3
*Klebsiella pneumoniae*	0	3
*Enterobacter cloacae*	0	1
*Pseudomonas aeruginosa*	0	3
*Moraxella catarrhalis*	2 (1)	1
*Mycoplasma pneumoniae*	38 (10)	28 (1)
*Chlamydophila pneumoniae*	0	4
Virus*	2	1
Unknown	55	69

*CTRX* Ceftriaxone, *ABPC/SBT* Ampicillin/sulbactam, *MSSA* Methicillin-sensitive *Staphylococcus aureus*. The number of cases with other pathogens detected is indicated in parentheses. Sputum examinations were not possible in 58 cases of the CTRX group and 69 cases of the ABPC/SBT group (*p* = 0.711). Missing data were in three patients of the CTRX group vs in seven of the ABPC/SBT group for blood culture examination (*p* = 0.343), one vs one for *Mycoplasma pneumoniae* and *Chlamydophila pneumoniae* antibody tests (*p* = 1.000), 22 vs 19 for urinary antigen tests of *Streptococcus pneumoniae* and *Legionella pneumophilia* serogroup 1 (*p* = 0.398). *Diagnosed by influenza virus antigen using throat or nasal swab specimens

### Safety and tolerability

All patients in the VPP population were evaluated for safety. Table 5 shows the adverse events probably related to the study drug, which were observed in 22 patients (25 events) in the CTRX group and 19 (20 events) in the ABPC/SBT group. Diarrhea was most frequently observed event in both groups. No patients in either group needed to change the treatment drug because of side effects.

**Table 5:** Adverse events possibly or probably related to the study drug

	CTRX (*n* = 124)	ABPC/SBT (*n* = 139)	*p*-value
Diarrhea	12	7	0.23
Rash	5	7	0.55
Elevated AST or ALT	4	4	1.00
Leukopenia	1	1	1.00
Nausea	1	1	1.00
Fever	1	0	0.48
Elevated creatine kinase	1	0	0.48
Total	25 (22 patients)	20 (19 patients)	

*CTRX* Ceftriaxone, *ABPC/SBT* Ampicillin/sulbactam, *AST* Aspartate aminotransferase, *ALT* Alanine aminotransferase

## Discussion

In the present study, the clinical effectiveness rates of CTRX and ABPC/SBT were compared in patients with CAP without risk factors for aspiration pneumonia. To our knowledge, this comparison has not been investigated in previous studies. Both antibiotics showed a high clinical effectiveness rate, and no significant differences were found between CTRX and ABPC/SBT at EOT (*p* = 0.072) in the VPP population. However, at day 7, the clinical effectiveness rate was higher for ABPC/SBT than for CTRX (*p* = 0.047). A similar result was obtained on days 4 and 7 in the MITT population (*p* = 0.018, 0.007).

It has been reported that there is no significant difference between β-lactam/β-lactamase inhibitor combinations and third-generation cephalosporins in the treatment of CAP [13–16]. However, most of those previous reports were retrospective studies, except for one that, unfortunately, did not undertake a direct comparison between β-lactam/β-lactamase inhibitor combinations and third-generation cephalosporins. Those studies included cases of aspiration pneumonia. For aspiration pneumonia, Hasegawa reported in a retrospective propensity score-matching analysis that mortality was not significantly different between ABPC/SBT and CTRX groups [17]. However, whether the two drugs show equivalent effectiveness in the treatment of pneumonia without aspiration risk remains unclear. To our knowledge, the present study is the first to compare the efficacy of CTRX and ABPC/SBT in the treatment of patients with CAP without risk factors for aspiration pneumonia.

The differences in efficacy between CTRX and ABPC/SBT in CAP treatment would be due to anaerobic bacteria, which are common causes of aspiration pneumonia [8–10]. However, anaerobic bacteria can also cause pneumonia in patients without apparent risk factors for aspiration pneumonia. In a previous study that carried out a bacterial floral analysis of 16S rRNA gene sequences in patients with CAP [29], 17.9% of detected bacteria in bronchoalveolar lavage fluid samples were anaerobic, even in patients without risk factors for aspiration. Although the authors stated that it was unclear whether anaerobic bacteria were the primary cause [29], their results suggest that anaerobic bacteria may cause pneumonia in some patients without risk factors for aspiration. These reasons might explain the difference in effectiveness rate in the early phase between CTRX and ABPC/SBT in the present study, because the susceptibility of anaerobic bacteria to third-generation cephalosporins is lower than that to β-lactam/β-lactamase inhibitor combinations [12].

At the time of launching our study, the ABPC/SBT dose was determined according to available drug information in Japan describing that no more than 3 g every 12 h should be administered [30]. Although this dose is lower than the most current standard dose of 3 g every 6 h [31], comparable clinical effectiveness was seen between the ABPC/SBT and CTRX groups, suggesting that a dose of 3 g ABPC/SBT every 12 h might be sufficient in CAP treatment. Recently, shortages in antibiotics have been increasingly discussed [32, 33]. In particular, shortages of penicillin and cephem antibiotics are becoming increasingly common. These problems have led to the use of broader-spectrum or more toxic antibiotics, and concerns about an increase in resistant bacteria and side effects due to inappropriate use have been raised. From the result of our study, treatment with 3 g ABPC/SBT every 12 h could be an alternative dose for patients with CAP if a shortage of ABPC/SBT becomes a serious problem.

In the present study, we examined the safety of CTRX and ABPC/SBT. The most frequent side effect of both treatments was diarrhea. Concerns have also been raised that antibiotics acting on anaerobic bacteria are associated with diarrhea [34–36]. However, in the present study, no significant difference in the frequency of diarrhea during antibiotic therapy with CTRX and ABPC/SBT was found. Rash and elevated liver enzymes were also observed at the same frequency in both treatment groups. Neither of these antibiotics is associated with life-threatening side effects, and no significant differences were observed in the frequency of any side effect between the CTRX and ABPC/SBT groups. Therefore, both antibiotics could be safely used for the treatment of CAP.

One of the strengths of our study was the quasi-randomized design. A trial using a true randomized design takes considerable time and effort to run, and this method may cause treatment delays and difficulties with patient recruitment in the emergency setting. Although selection bias could be present in a quasi-randomized study, no strong evidence has yet shown that selection bias in emergency settings occurs more often with quasi-randomization than with true randomization [37]. Quasi-randomization could sometimes be an appropriate method in emergency settings.

The present study had several limitations. First, there was uncertainty about the effect size. The difference between groups could potentially be smaller or non-existent. Second, this was a single-center trial. Because the background characteristics of patients would differ between hospitals, the severity of pneumonia would be different. Therefore, whether the clinical differences observed between the two antibiotics in the present study can be generalized needs to be investigated in a follow-up study. Third, to judge whether the patient had aspiration pneumonia, risk factors for aspiration were evaluated, but not swallowing function or silent aspiration at night. Some cases of pneumonia in the present study might thus have been caused by silent aspiration of oral bacteria. Distinguishing pneumonia caused by oral anaerobic bacteria from that caused by other bacteria by evaluating only the risk factors for aspiration was difficult. However, because it is not routine in clinical practice to evaluate swallowing function to judge whether a patient has aspiration pneumonia, the results of the present study can be considered at least somewhat reflective of actual clinical practice.

## Conclusion

In conclusion, no significant difference was observed between ABPC/SBT and CTRX at EOT, but ABPC/SBT might be more effective in the early phase of treatment.

## Data Availability

The datasets used and/or analyzed during the current study are available from the corresponding author on reasonable request.

## Abbreviations

ABPC/SBT: Ampicillin/sulbactam
BT: Body temperature
CAP: Community-acquired pneumonia
CRP: C-reactive protein
CTRX: Ceftriaxone
EOS: End of study
EOT: End of treatment
MITT: Modified intention-to-treat
MSSA: Methicillin-sensitive *Staphylococcus aureus*
PSI: Pneumonia severity index
VPP: Validated per-protocol
WBC: White blood cell

## References

[1] Yanagihara K, Kadota J, Aoki N, Matsumoto T, Yoshida M, Yagisawa M (2015). Nationwide surveillance of bacterial respiratory pathogens conducted by the surveillance committee of Japanese Society of Chemotherapy, the Japanese Association for Infectious Diseases, and the Japanese Society for Clinical Microbiology in 2010: general view of the pathogens’ antibacterial susceptibility. J Infect Chemother.

[2] Niki Y, Hanaki H, Yagisawa M, Kohno S, Aoki N, Watanabe A (2008). The first nationwide surveillance of bacterial respiratory pathogens conducted by the Japanese Society of Chemotherapy. Part 1: a general view of antibacterial susceptibility. J Infect Chemother.

[3] American Thoracic Society, Infectious Diseases Society of America. Guidelines for the management of adults with hospital-acquired, ventilator-associated, and healthcare-associated pneumonia. Am J Respir Crit Care Med. 2005;171(4):388–416. 10.1164/rccm.200405-644ST 15699079

[4] Kohno S, Imamura Y, Shindo Y, Seki M, Ishida T, Teramoto S (2013). Clinical practice guidelines for nursing- and healthcare-associated pneumonia (NHCAP) [complete translation]. Respir Investig.

[5] ERS Task Force Report (1998). Guidelines for management of adult community-acquired lower respiratory tract infections. European Respiratory Society. Eur Respir J.

[6] Bartlett JG, Dowell SF, Mandell LA, File TM, Musher DM, Fine MJ (2000). Practice guidelines for the management of community-acquired pneumonia in adults. Infectious Diseases Society of America. Clin Infect Dis.

[7] Niederman MS, Mandell LA, Anzueto A, Bass JB, Broughton WA, Campbell GD (2001). Guidelines for the management of adults with community-acquired pneumonia. Diagnosis, assessment of severity, antimicrobial therapy, and prevention. Am J Respir Crit Care Med.

[8] Bartlett JG, Gorbach SL (1975). The triple threat of aspiration pneumonia. Chest..

[9] Cesar L, Gonzalez C, Calia FM (1975). Bacteriologic flora of aspiration-induced pulmonary infections. Arch Intern Med.

[10] Tokuyasu H, Harada T, Watanabe E, Okazaki R, Touge H, Kawasaki Y (2009). Effectiveness of meropenem for the treatment of aspiration pneumonia in elderly patients. Intern Med.

[11] Bartlett JG (2013). How important are anaerobic bacteria in aspiration pneumonia: when should they be treated and what is optimal therapy. Infect Dis Clin N Am.

[12] Citron DM, Tyrrell KL, Merriam CV, Goldstein EJ (2010). In vitro activity of ceftaroline against 623 diverse strains of anaerobic bacteria. Antimicrob Agents Chemother.

[13] de Klerk GJ, van Steijn JH, Lobatto S, Jaspers CA, van Veldhuizen WC, Hensing CA (1999). A randomised, multicentre study of ceftriaxone versus standard therapy in the treatment of lower respiratory tract infections. Int J Antimicrob Agents.

[14] Xaba SN, Greeff O, Becker P (2014). Determinants, outcomes and costs of ceftriaxone v. amoxicillin-clavulanate in the treatment of community-acquired pneumonia at Witbank hospital. S Afr Med J.

[15] Sanchez ME, Gomez J, Gomez Vargas J, Banos V, Ruiz Gomez J, Munoz L (1998). Prospective and comparative study between cefuroxime, ceftriaxone and amoxicillin-clavulanic acid in the treatment of community-acquired pneumonia. Rev Esp Quimioter.

[16] Terahara F, Kisa K, Yamada K, Yokokawa Y, Saito S (2017). Efficacy of Ceftriaxone in Aspiration Pneumonia Propensity Score Matched Retrospective Observational Study, Compared with Sulbactam/ampicillin. Iryo Yakugaku (Japanese J Pharm Health Care Sci).

[17] Hasegawa S, Shiraishi A, Yaegashi M, Hosokawa N, Morimoto K, Mori T (2019). Ceftriaxone versus ampicillin/sulbactam for the treatment of aspiration-associated pneumonia in adults. J Comp Eff Res.

[18] Torres A, Garau J, Arvis P, Carlet J, Choudhri S, Kureishi A (2008). Moxifloxacin monotherapy is effective in hospitalized patients with community-acquired pneumonia: the MOTIV study--a randomized clinical trial. Clin Infect Dis.

[19] Ito I, Kadowaki S, Tanabe N, Haruna A, Kase M, Yasutomo Y (2010). Tazobactam/piperacillin for moderate-to-severe pneumonia in patients with risk for aspiration: comparison with imipenem/cilastatin. Pulm Pharmacol Ther.

[20] Fine MJ, Auble TE, Yealy DM, Hanusa BH, Weissfeld LA, Singer DE (1997). A prediction rule to identify low-risk patients with community-acquired pneumonia. N Engl J Med.

[21] Hu X, Lee JS, Pianosi PT, Ryu JH (2015). Aspiration-related pulmonary syndromes. Chest..

[22] van der Maarel-Wierink CD, Vanobbergen JN, Bronkhorst EM, Schols JM, de Baat C (2011). Risk factors for aspiration pneumonia in frail older people: a systematic literature review. J Am Med Dir Assoc.

[23] Adnet F, Baud F (1996). Relation between Glasgow Coma Scale and aspiration pneumonia. Lancet.

[24] Priefer BA, Robbins J (1997). Eating changes in mild-stage Alzheimer’s disease: a pilot study. Dysphagia..

[25] Thomas FJ, Wiles CM (1999). Dysphagia and nutritional status in multiple sclerosis. J Neurol.

[26] O'Neill OM, Johnston BT, Coleman HG (2013). Achalasia: a review of clinical diagnosis, epidemiology, treatment and outcomes. World J Gastroenterol.

[27] Enzinger PC, Mayer RJ (2003). Esophageal cancer. N Engl J Med.

[28] Dietrich ES, Joseph U, Vogel F, Howaldt S, Kullmann KH, Frank U (1999). Cost-effectiveness of ceftriaxone 1 g vs second-generation cephalosporins in the treatment of pneumonia in general medical wards in Germany. Infection..

[29] Akata K, Yatera K, Yamasaki K, Kawanami T, Naito K, Noguchi S (2016). The significance of oral streptococci in patients with pneumonia with risk factors for aspiration: the bacterial floral analysis of 16S ribosomal RNA gene using bronchoalveolar lavage fluid. BMC Pulm Med.

[30] Kadowaki M, Demura Y, Mizuno S, Uesaka D, Ameshima S, Miyamori I (2005). Reappraisal of clindamycin IV monotherapy for treatment of mild-to-moderate aspiration pneumonia in elderly patients. Chest..

[31] Kohno S, Tateda K, Mikamo H, Kadota J, Niki Y, Itamura R (2015). Efficacy and safety of intravenous sulbactam/ampicillin 3 g 4 times daily in Japanese adults with moderate to severe community-acquired pneumonia: a multicenter, open-label, uncontrolled study. J Infect Chemother.

[32] Quadri F, Mazer-Amirshahi M, Fox ER, Hawley KL, Pines JM, Zocchi MS (2015). Antibacterial drug shortages from 2001 to 2013: implications for clinical practice. Clin Infect Dis.

[33] Gundlapalli AV, Beekmann SE, Graham DR, Polgreen PM (2018). Antimicrobial Agent Shortages: The New Norm for Infectious Diseases Physicians. Open Forum Infect Dis.

[34] Wistrom J, Norrby SR, Myhre EB, Eriksson S, Granstrom G, Lagergren L (2001). Frequency of antibiotic-associated diarrhoea in 2462 antibiotic-treated hospitalized patients: a prospective study. J Antimicrob Chemother.

[35] McFarland LV (2008). Antibiotic-associated diarrhea: epidemiology, trends and treatment. Future Microbiol.

[36] Owens RC, Donskey CJ, Gaynes RP, Loo VG, Muto CA (2008). Antimicrobial-associated risk factors for Clostridium difficile infection. Clin Infect Dis.

[37] Corbett MS, Moe-Byrne T, Oddie S, McGuire W (2016). Randomization methods in emergency setting trials: a descriptive review. Res Synth Methods.

